# Development and validation of the Maudsley Modified Patient Health Questionnaire (MM-PHQ-9)

**DOI:** 10.1192/bjo.2021.953

**Published:** 2021-07-02

**Authors:** Phillippa Harrison, Syndi Walton, Diede Fennema, Suqian Duan, Tanja Jaeckle, Kimberley Goldsmith, Ewan Carr, Mark Ashworth, Allan. H. Young, Roland Zahn

**Affiliations:** Department of Psychological Medicine, Centre for Affective Disorders, Institute of Psychiatry, Psychology & Neuroscience, King's College London, UK; Department of Psychological Medicine, Centre for Affective Disorders, Institute of Psychiatry, Psychology & Neuroscience, King's College London, UK; Department of Psychological Medicine, Centre for Affective Disorders, Institute of Psychiatry, Psychology & Neuroscience, King's College London, UK; Department of Psychological Medicine, Centre for Affective Disorders, Institute of Psychiatry, Psychology & Neuroscience, King's College London, UK; Department of Psychological Medicine, Centre for Affective Disorders, Institute of Psychiatry, Psychology & Neuroscience, King's College London, UK; Department of Biostatistics and Health Informatics, Institute of Psychiatry, Psychology & Neuroscience, King's College London, UK; Department of Biostatistics and Health Informatics, Institute of Psychiatry, Psychology & Neuroscience, King's College London, UK; School of Population Health and Environmental Sciences, King's College London, UK; Department of Psychological Medicine, Centre for Affective Disorders, Institute of Psychiatry, Psychology & Neuroscience, King's College London, UK; and National Service for Affective Disorders, South London and Maudsley NHS Foundation Trust, UK; Department of Psychological Medicine, Centre for Affective Disorders, Institute of Psychiatry, Psychology & Neuroscience, King's College London, UK; and National Service for Affective Disorders, South London and Maudsley NHS Foundation Trust, UK

**Keywords:** Depression, measurement, digital health, antidepressants, primary care

## Abstract

**Background:**

The Patient Health Questionnaire-9 (PHQ-9) is a widely used measure of depression in primary care. It was, however, originally designed as a diagnostic screening tool, and not for measuring change in response to antidepressant treatment. Although the Quick Inventory of Depressive Symptomology (QIDS-SR-16) has been extensively validated for outcome measurement, it is poorly adopted in UK primary care, and, although free for clinicians, has licensing restrictions for healthcare organisation use.

**Aims:**

We aimed to develop a modified version of the PHQ-9, the Maudsley Modified PHQ-9 (MM-PHQ-9), for tracking symptom changes in primary care. We tested the measure's validity, reliability and factor structure.

**Method:**

A sample of 121 participants was recruited across three studies, and comprised 78 participants with major depressive disorder and 43 controls. MM-PHQ-9 scores were compared with the QIDS-SR-16 and Clinical Global Impressions improvement scale, for concurrent validity. Internal consistency of the scale was assessed, and principal component analysis was conducted to determine the items’ factor structure.

**Results:**

The MM-PHQ-9 demonstrated good concurrent validity with the QIDS-SR-16, and excellent internal consistency. Sensitivity to change over a 14-week period was *d* = 0.41 compared with *d* = 0.61 on the QIDS-SR-16. Concurrent validity between the paper and mobile app versions of the MM-PHQ-9 was *r* = 0.67.

**Conclusions:**

These results indicate that the MM-PHQ-9 is a valid and reliable measure of depressive symptoms in paper and mobile app format, although further validation is required. The measure was sensitive to change, demonstrating suitability for use in routine outcome assessment.

Measurement of depressive symptoms is important for determining change over time, such as when assessing response to treatment. The late 20th century brought a rapid increase in the number of rating scales for depression, both clinician- and self-rated.^1^ A commonly used clinician-rated scale in depression treatment research is the Hamilton Rating Scale for Depression (HRSD^2,3^), and a commonly used self-rated scale is the Beck Depression Inventory-II,^4^ although it is now unaffordable in some routine clinical settings. The advantages and disadvantages of clinician- versus self-rated scales have been heavily debated, with some clear evidence for discrepancies in scoring between clinicians and patients.^5^ Clinician-rated scales are often hailed as the gold standard of rating scales, but there are many advantages to self-rated scales. One compelling advantage of self-rated measures of depression is their efficiency, especially for use over repeated assessments, requiring less clinician time and, hence, being more cost-effective. The Patient Health Questionnaire-9 (PHQ-9^6^) has demonstrated high validity as a self-reported outcome scale in primary care, and is widely used because of its lack of licensing restrictions and brevity. However, the PHQ-9 has some limitations relating to its intervals, specificity of symptoms for depression and predictive ability of symptoms.

The PHQ-9 was devised as a diagnostic screening tool using DSM-IV criteria.^7^ In line with DSM-IV criteria for depression diagnoses, the PHQ-9 assesses depressive symptoms over the past 2 weeks. Yet, rating scales with 1-week intervals are more sensitive to change, making them better suited to treatment or research settings.^8^ Furthermore, when asked to reflect over a longer period, many patients find it hard to accurately remember their symptoms, which can create unreliable data.^9^

Furthermore, the PHQ-9, like the DSM-IV/5, collapses ‘feeling down and depressed’ and ‘feeling hopeless’ into one item. Although this may be acceptable for diagnostic purposes, it is problematic for measuring severity. Hopelessness and depressed mood have been found to load onto separate factors in major depressive disorder (MDD), demonstrating the importance of hopelessness in patients who are suicidal,^10^ whereas depressed mood is present across the whole range of depression severity. Furthermore, hopelessness is a key symptom in attributional models of depression where it plays a distinct role from depressed mood.^11^

Previous research has shown somatic symptoms to be poor predictors of remission following antidepressant treatment.^12^ In the Sequenced Treatment Alternatives to Relieve Depression trial, MDD remission was predicted by early improvement in mainly non-somatic symptoms, such as sad mood, for participants taking citalopram.^12^ Research has shown somatic symptoms to vary over time with no intervention, creating an unstable factor structure, whereas non-somatic symptoms, such as depressed mood, remain fairly constant.^13^ Furthermore, somatic symptoms were not predictive of future symptoms, whereas non-somatic symptoms were capable of predicting both somatic and non-somatic future symptoms, hence being more suitable for tracking treatment change.^13^ Additionally, somatic symptoms such as appetite and sleep changes can be a result of medication side-effects rather than antidepressant effects.

Psychomotor activity is included in the PHQ-9 as a depressive symptom, but has not been found to be a consistent symptom of MDD, and was shown to be sensitive to selective serotonin reuptake inhibitor (SSRI)-induced change only in severe depression.^14^ A further study found that Surinam Dutch men were more likely to score lower on psychomotor activity than other Dutch men of the same depression severity,^15^ indicating that different cultural backgrounds can influence likelihood to endorse this item. Huang et al also reported a cultural bias when testing Chinese Americans, who were more than twice as likely to endorse psychomotor activity changes as other cultural groups.^16^ Therefore, differences in PHQ-9 scoring by different cultural groups cannot be fully attributed to differences in depression. Furthermore, patients often overlook the criterion that psychomotor activity should be observed by others to be scored as ‘present’, resulting in an exaggerated PHQ-9 score.

Another weakness is that the PHQ-9 includes the item ‘feeling bad about yourself or that you are a failure, or have let yourself or your family down’, which encompasses both self-worth and guilt despite clear evidence that low self-worth is a more consistent symptom than guilt.^17–19^ Therefore, the separation of low self-worth and guilt could improve the scale's sensitivity to change in patients experiencing self-blame.

Finally, the PHQ-9 includes no anxiety-related symptoms following the original separation of the Primary Care Evaluation of Mental Disorders (PRIME-MD) into the PHQ-9 for depressive symptoms and the Generalised Anxiety Disorder Questionnaire (GAD-7) for anxiety symptoms. However, the DSM-5 has now incorporated anxiety with the anxious distress specifier for MDD because of its prognostic implications.^7^ Furthermore, there is evidence that psychological aspects of anxiety are good measures of SSRI response in severe and non-severe MDD.^14^

Hence, despite being a useful outcome measure, there are some weaknesses of the PHQ-9 that hinder its accuracy for measuring change in symptoms over time. It is important to recognise that the PHQ-9 is widely used as a severity measure rather than a diagnostic measure as originally developed, so the timescales and symptoms presented should be tailored to accurately measure the changes in depressive symptoms.

## Aims

In this paper, we developed the Maudsley Modified PHQ-9 (MM-PHQ-9) to address the described weaknesses of the PHQ-9 as a measure of change, and present initial data on its validity and reliability. The long-term goal is to provide a freely available tool for tracking antidepressant response in primary care settings. Our specific aims and hypotheses were as follows:

- To determine the concurrent validity of the MM-PHQ-9 with pre-existing self-report measures of depressive symptoms (Quick Inventory of Depressive Symptomology (QIDS-SR-16^20^) at baseline. We hypothesised that the MM-PHQ-9 would correlate with the QIDS-SR-16 as a measure of the common construct of depressive severity. However, higher concurrent validity with the Very Quick Inventory of Depressive Symptomatology (VQIDS-SR5^21^) was hypothesised than with the QIDS-SR-16, as the VQIDS-SR5 excludes somatic items.
- To determine the concurrent validity of baseline MM-PHQ-9 when presented in a mobile app compared with a paper/online format. We hypothesised that MM-PHQ-9 scores would be correlated when presented in both formats.
- To determine the sensitivity, specificity, and positive and negative predictive values of baseline MM-PHQ-9. We hypothesised that the MM-PHQ-9 would have high specificity and sensitivity, similar to the original PHQ-9.
- To determine the sensitivity to change from baseline to follow-up in depressive symptoms of the MM-PHQ-9 by comparing it with standard measures that can detect change in symptoms: the self-rated Clinical Global Impression Improvement Scale (CGI-Improvement^22^) and change in QIDS-SR-16 scores. We hypothesised that the MM-PHQ-9 would be comparable to the CGI-Improvement and QIDS-SR-16 in its ability to detect changes in symptoms.
- To test the inter-item correlation of baseline MM-PHQ-9 to evaluate its reliability as an assessment of depression. MM-PHQ-9 items were hypothesised to correlate with one another, as all reflecting the common construct of depression severity.
- To investigate the underlying factor structure of the MM-PHQ-9 scale, using principal component analysis on baseline scores. We hypothesised that the additional items added will load onto a common depression factor with the existing depression items.

## Method

### Design

Secondary data analysis was conducted on data from the Antidepressant Advisor Study (ADeSS), Neurofeedback in Depression (NeuroMooD)and Sadness is Good (SiG) studies (see Table 1). The ADeSS research portfolio comprised three studies on MDD. ADeSS study 1 was a feasibility trial of a decision-support tool to assist general practitioners with antidepressant prescribing for treatment-resistant MDD.^23^ ADeSS study 2 was an online recruitment and survey study to investigate combinations of patient factors associated with response to treatments for depression. Participants followed a similar study process to ADeSS study 1, without the intervention. ADeSS study 3 aimed to provide the proof of concept for using functional magnetic resonance imaging (fMRI) biomarkers to prospectively predict which patients would not benefit from standard SSRI treatment, and comparing fMRI measures with participants without depression.^23^ For the purpose of this analysis, participants from ADeSS studies 1 and 2 were included in the MDD sample, and controls from ADeSS study 3 were included in the control sample. The NeuroMooD trial examined a novel real-time fMRI neurofeedback method in MDD, and also included a case–control cross-sectional study recruiting healthy controls.^24^ Both MDD and control samples were included in this analysis. The SiG study aimed to use a method of emotion reappraisal to train healthy participants with a tendency for self-blame to convert their self-blame to sadness, as a more adaptive emotion.

**Table 1:** Descriptive statistics of gender, age and years in education for the clinical and control samples

Study	*n*	Gender, % female	Age, years, mean (s.d.)	Years in education, mean (s.d.)
Clinical				
ADeSS study 1	22	81.8	51.45 (15.12)	13.91 (2.83)
ADeSS study 2	51	98	29.19 (9.86)	15.76 (3.44)
NeuroMooD	5	100	36.60 (6.84)	17.00 (3.08)
Control				
SiG	20	−	−	−
ADeSS study 3	5	80	35.60 (14.78)	17.80 (2.59)
NeuroMooD	18	83.3	42.00 (16.16)	16.61 (2.64)

No demographic data was available for the SiG study. ADeSS, Antidepressant Advisor Study; NeuroMooD, Neurofeedback in Depression trial; SiG, Sadness is Good study.

ADeSS study 1, ADeSS study 2 and NeuroMooD participants formed the clinical sample for comparing the MM-PHQ-9 with the QIDS-SR-16 for aim (a), and ADeSS study 3 and NeuroMooD controls formed the control sample. ADeSS studies 1 and 2 formed the clinical sample for comparing the MM-PHQ-9 with the VQIDS-SR5. For aim (b), a subsample of participants from ADeSS studies 1 and 2 completed a mobile app version of the MM-PHQ-9, which was compared with the paper version. For aim (c), all participants’ MM-PHQ-9 scores were assessed for sensitivity and specificity. For aim (d), change in MM-PHQ-9 score was compared with CGI-Improvement and QIDS-SR-16 change scores in a subsample of participants from ADeSS studies 1 and 2 and all NeuroMooD study participants. For aim (e), MM-PHQ-9 item scores were analysed for clinical sample inter-item correlation for ADeSS study 1, ADeSS study 2 and NeuroMooD study participants, and scores of ADeSS study 3 controls and NeuroMooD study controls were analysed for control sample reliability. For aim (f), principal component analysis was conducted on MM-PHQ-9 item scores of participants from ADeSS studies 1 and 2.

### Participants

Participants from the three studies who had completed the MM-PHQ-9 were included in our analysis (ADeSS: *n* = 73 MDD and *n* = 5 non-MDD control participants; SiG: *n* = 20 healthy controls; NeuroMooD: *n* = 5 MDD and 18 healthy control participants), resulting in a total sample of 121 individuals, with 78 MDD and 43 non-MDD control participants. We had missing demographic data for the 20 SiG participants. The remaining sample of 101 participants was 91.1% female (7.9% male, 1% non-binary), with a mean age of 37.24 (s.d. 15.35) and mean years of education of 15.67 (s.d. 3.26). All participants completed baseline assessments.

Inclusion and exclusion criteria varied for each study and are provided in more detail in the relevant study publications. For the purpose of the current analysis, common inclusion criteria across studies for the MDD sample were as follows: age ≥18 years; proficient in English; and at least moderately severe major depressive syndrome on the PHQ-9 (score ≥15; ADeSS studies 1 and 2) or a diagnosis of recurrent MDD according to the DSM-5^7^ (NeuroMooD). Exclusion criteria for the MDD sample were as follows: unstable medical condition, neurological condition, history of manic/hypomanic episodes, history of schizophreniform symptoms or schizophrenia and current/recent drug misuse.

For the non-MDD control sample, common inclusion criteria across studies were age ≥18 years and proficient in English. Exclusion criteria were a diagnosis of MDD or current major depressive syndrome, and bipolar or psychotic disorders.

Key differences in inclusion criteria were that participants from ADeSS studies 1 and 2 were required to be taking an antidepressant at baseline (or had in past 2 months), whereas this was not the case for NeuroMooD study participants. Participants from ADeSS studies 1 and 2 were permitted to undergo psychotherapy during study participation, but this was an exclusion criteria for the NeuroMooD study. Diagnostic procedures are detailed in the Supplementary Methods available at https://doi.org/10.1192/bjo.2021.953.

### Measures

#### MM-PHQ-9

The MM-PHQ-9 is an adapted version of the PHQ-9, designed to assess depressive symptoms.^6^ Amendments were made to reflect evidence-based knowledge of depressive symptoms: questions 2 and 3 were separated to represent depressed mood and hopelessness separately. Somatic symptoms (sleep, appetite) and the psychomotor item were omitted. Question 5 was added to assess self-blaming emotions, and was validated^17^ to detect self-blaming emotions in 60% of patients with MDD. This study found guilt to dissociate from low self-worth, and therefore question 6 was simplified. Intervals for symptom assessment were changed from biweekly to weekly. This necessitated changing the wording of the scale anchors to ‘some days’ instead of ‘several days’ and ‘every day’ instead of ‘nearly every day’. As with the PHQ-9, the scale comprises 9 items and the total score is derived from summing item scores, with a total range of 0–27.

#### QIDS-SR-16

The QIDS-SR-16 is a self-rated measurement of depressive symptom severity. The measure has 16 items that assess a range of depressive symptoms, including mood and somatic-related symptoms. The measure is scored by summing the highest scoring item from items 1–4; item 5; the highest scoring item from items 6–9; items 10, 11, 12, 13 and 14; and the highest scoring item from items 15 and 16. It has good internal consistency (*α* = 0.86) and is highly correlated with the HRSD-24 (*r* = 0.86).

#### VQIDS-SR5

The VQIDS-SR5 is a shorter, five-item version of the QIDS-SR-16 that measures the core symptoms of depression: sad mood, self-outlook, involvement, fatigue and psychomotor slowing. These items reflect those included in the HRSD-6, with the removal of anxiety.^25^ The measure has a reduced focus on somatic symptoms compared with the QIDS-SR-16, and so was a useful comparison in our study against the MM-PHQ-9. The scale has good internal consistency (*α* = 0.67−0.81), and strong concurrent validity with the QIDS-SR-16 (*r* = 0.90).

#### CGI-Improvement

The CGI-Improvement (self-rated) is a self-rated measurement scale of change in depression over time, based on a combination of symptoms and functioning level. There are seven ordinal categories, from one (very much improved) to seven (very much worse).

### Interventions

ADeSS study 1 participants had their antidepressant treatment reviewed by their general practitioner within 2 weeks of baseline, and potentially changed to another antidepressant or higher dose, continuing to be changed an unlimited number of times over the 14-week study duration. Follow-up occurred 15–18 weeks after baseline. ADeSS study 2 participants received no intervention and were followed up 15–18 weeks after baseline. NeuroMooD study participants received three neurofeedback intervention sessions over 5 weeks, and were required to remain on the same antidepressant and dose. Follow-up occurred up to 52 days after baseline. No control participants from ADeSS study 3, the NeuroMooD study or SiG study received any interventions or were followed up on.

### Procedure

The authors assert that all procedures contributing to this work comply with the ethical standards of the relevant national and institutional committees on human experimentation and with the Helsinki Declaration of 1975, as revised in 2008. All procedures involving human participants were approved by the Camberwell & St Giles NHS Research Ethics Committee (ADeSS study reference number 17/LO/2074; NeuroMooD study reference number 15/LO/0577) and Psychiatry, Nursing and Midwifery Research Ethics Subcommittee at King's College London (SiG study reference number HR-17/18-6151). Written informed consent was obtained from all participants for the studies and for their data to be used for future research. All participants completed a paper or online version of the MM-PHQ-9 at baseline, in addition to other measures, as part of their study participation. A subsample of participants with MDD in ADeSS studies 1 and 2 completed a mobile app version (MooDoC version 1.67.0 for Android, Alloc Modulo Ltd, London, UK) of the MM-PHQ-9 (https://play.google.com/store/apps/details?id=com.allocmodulo&hl=en_GB&gl=US) within approximately 2 weeks of baseline. Participants with MDD completed paper or online follow-up assessments. The ADeSS studies were registered with Clinicaltrials.gov, under registration numbers NCT03628027 (study 1) and NCT04342299 (study 3); the NeuroMooD study was registered with the ISRCTN Registry, under registration number ISRCTN10526888. ADeSS study 2 and SiG studies were not pre-registered.

### Analysis

Data analysis was conducted with SPSS for Windows version 26 (IBM^26^). Because not all studies administered all of the measures of interest, there were varying sample sizes for different analyses. The distribution of the data was assessed with the Shapiro–Wilk test of normality, and non-parametric adaptations were applied to satisfy the assumptions of a normal distribution.

The analysis proceeded in six stages. First, we aimed to determine the concurrent validity of the MM-PHQ-9 at baseline, with the QIDS-SR-16,^20^ and the VQIDS-SR5,^21^ by conducting correlations between the MM-PHQ-9 and each scale. Second, we aimed to determine the concurrent validity of the MM-PHQ-9 at baseline when presented in a mobile app compared with an online and paper format, by conducting correlations between the baseline MM-PHQ-9 and the initial mobile app MM-PHQ-9. Third, we aimed to determine the sensitivity, specificity positive and negative predictive values of the MM-PHQ-9 by calculating the proportion of participants from the MDD and control samples with scores >9 versus scores ≤9. This was based on the PHQ-9 cut-off criteria of a score of >9 being categorised as moderate depressive symptoms. Fourth, we aimed to determine the sensitivity to change in depressive symptoms of the MM-PHQ-9 by comparing it with the CGI-Improvement and change in QIDS-SR-16 scores, by conducting paired sample *t*-tests between MM-PHQ-9 baseline and follow-up scores, and QIDS-SR-16 scores. Cohen's *d* effect sizes were calculated and compared for each measure, using the formula: *d* = mean change score/s.d. change score. A correlation was conducted between change on the MM-PHQ-9 from baseline to follow-up and CGI-Improvement ratings. Fifth, we aimed to test the reliability of the MM-PHQ-9 at baseline, to assess the presence and the severity of depression, by conducting a test of internal consistency of the MM-PHQ-9 items and deriving Cronbach's alpha, and we assessed the strength of inter-item correlations. Finally, we aimed to use principal component analysis to investigate the underlying factor structure of the MM-PHQ-9, including the new items. Because of correlated factors, an Oblimin rotation with Kaiser normalisation was applied to simplify the factor structure. Factors with Eigenvalues above 1 were retained as true factors. A cut-off point of ≥0.7 was applied to item loadings, to be recognised as loading onto a factor.^27^

## Results

### Available data

Forty-eight participants from the MDD sample had available follow-up data that was collected in the required time period; follow-up data was not collected for the control sample. Thirty-four participants from ADeSS studies 1 and 2 provided MM-PHQ-9 data via a mobile app. Demographic data was available for 101 participants (Table 1). The MDD group in ADeSS study 1 had a significantly higher mean age (mean 51.45 years, s.d. 15.12 years) than the MDD groups in ADeSS study 2 (mean 29.19 years, s.d. 9.86 years) and the NeuroMooD study (mean 36.60 years, s.d. 6.84 years) (*F*(2,72) = 28.16, *P* < 0.001). A *post hoc* Bonferroni test showed this difference to be significant between ADeSS study 1 and ADeSS study 2 (*t*(21) = 22.27, *P* < 0.001) and the NeuroMooD study (*t*(4) = 14.86, *P* = 0.034).

### MM-PHQ-9 and QIDS-SR-16 scores

The mean baseline MM-PHQ-9 score for the MDD sample was 17.23 (see Table 2), which is typically considered as a moderately severe symptom level on the standard PHQ-9. The mean baseline QIDS-SR-16 score was also 17.23. In comparison, the mean baseline MM-PHQ-9 total for the control sample was 3.67, which is usually considered as no/minimal symptoms on the standard PHQ-9. The mean baseline QIDS-SR-16 score for the control sample was 3.09.

**Table 2:** MM-PHQ-9 and QIDS-SR-16 total score mean, s.d. and range

	*n*	MM-PHQ-9			*n*	QIDS-SR-16		
		Mean	s.d.	Range		Mean	s.d.	Range
MDD baseline	78	17.23	4.49	7–26	78	17.23	3.46	7–24
MDD follow-up	48	14.40	6.96	1–26	47	13.89	5.78	0–24
Control baseline	43	3.67	3.43	0–9	23	3.09	1.41	0–5

MM-PHQ-9, Maudsley-modified Patient Health Questionnaire-9; QIDS-SR-16, Quick Inventory of Depressive Symptomatology Self-rated-16 item version; MDD, major depressive disorder.

At follow-up, the mean MM-PHQ-9 score for the MDD sample was 14.40, which falls into the moderate symptom category on the PHQ-9. This produced a mean decrease in symptoms of 12% (s.d. 39.51, range from 90% increase in symptoms to 95% decrease). The mean QIDS-SR-16 follow-up score was 13.89, which produced a mean decrease in symptoms of 18% (s.d. 30.01, range from 38% increase to 100% decrease).

### Distribution of MM-PHQ-9 and QIDS-SR-16 scores and concurrent validity

The distribution of MDD and control MM-PHQ-9 total scores combined was examined with a Shapiro–Wilk test of normality, and showed a significantly non-normal distribution (*Shapiro–Wilk* (101) = 0.92, *P <* 0.001). Similarly, a Shapiro–Wilk test revealed that the QIDS-SR-16 total score was not normally distributed (*Shapiro–Wilk* (101) = 0.88, *P <* 0.001). For the MDD sample, the distribution of the MM-PHQ-9 total scores were normally distributed (*Shapiro–Wilk* (78) = 0.93, *P =* 0.096), as were the QIDS-SR-16 total scores (*Shapiro–Wilk* (78) = 0.97, *P =* 0.113).

For the control sample, the distribution of the MM-PHQ-9 total scores (*Shapiro–Wilk* (23) = 0.77, *P <* 0.001) and QIDS-SR-16 total scores (*Shapiro–Wilk* (23) = 0.89, *P =* 0.016) were significantly non-normally distributed (see Table 3 for all concurrent validity statistics).

**Table 3:** Concurrent validity of measures with the MM-PHQ-9

	*n*	*r*	*d.f.*	*P-*value
QIDS-SR-16				
Total	101	0.83	101	<0.01
MDD	78	0.65	78	<0.01
Control	23	0.67	23	<0.001
VQIDS-SR5				
MDD	73	0.63	73	<0.001
Mobile app MM-PHQ-9				
MDD	34	0.67	32	<0.001

MM-PHQ-9, Maudsley-modified Patient Health Questionnaire-9; QIDS-SR-16, Quick Inventory for Depressive Symptomatology Self-rated 16 item version; MDD, major depressive disorder; VQIDS-SR5, Very Quick Inventory of Depressive Symptomatology – Self-Report.

### Relationship between MM-PHQ-9 and QIDS-SR-16 scores

A Spearman's correlation was conducted on the relationship between the MM-PHQ-9 and QIDS-SR-16 total scores at baseline. The analysis showed there to be high concurrent validity between the MM-PHQ-9 and QIDS-SR-16 in the total sample. A Pearson's correlation was conducted on the MDD sample only, and showed there to be moderate concurrent validity between the MM-PHQ-9 and QIDS-SR-16 total scores. This relationship was of similar strength for controls on whom a Spearman's correlation was conducted.

### Relationship between MM-PHQ-9 and VQIDS-SR5 scores

For a subsample of the MDD sample with available data, the VQIDS-SR5 total score was calculated. A Spearman's correlation showed the concurrent validity between VQIDS-SR5 and MM-PHQ-9 total scores to be similar to that of the full QIDS-SR-16.

### Mobile app MM-PHQ-9 score

There was a moderate correlation between participants’ baseline MM-PHQ-9 total score and their mobile app MM-PHQ-9 total score, indicating good concurrent validity between the two formats.

### Sensitivity and specificity

The sensitivity of the MM-PHQ-9 cut-off score of ≥10 was 98.7% (true positives: *n* = 77 out of 78 participants with MDD; Supplementary Fig. 1 and Table 4), with a specificity of 100.0% (true negatives: *n* = 43 out of 43 control participants), positive predictive value of 100.0% (true positives: *n* = 77 out of the sum of True and False Positives (*n* = 77)) and negative predictive value of 97.7% (true negatives: *n* = 43 out of the sum of true and false negatives (*n* = 44)). As the prevalence of MDD in our sample was much higher than one would expect in a naturalistic sample, we also calculated predictive values adjusted for 15% MDD prevalence, to illustrate how these results could translate to future samples (positive predictive value of 100% and negative predictive value of 99.77%).

### Sensitivity to change

One participant with QIDS-SR-16 data was excluded from this analysis because of a missing item on the QIDS-SR-16 at follow-up. For those in the MDD sample with follow-up data available (MM-PHQ-9 *n* = 48; QIDS-SR-16 *n* = 47), paired *t*-tests were conducted between baseline and follow-up MM-PHQ-9 and QIDS-SR-16 scores. Change in MM-PHQ-9 scores was significant (*t*(47) = 2.82, *P* = 0.007), with a small effect size (Cohen's *d* = 0.41). Change in QIDS-SR-16 scores was also significant (*t*(46) = 4.20, *P* < 0.001), but with a medium effect size (Cohen's *d* = 0.61).

There was also a moderate significant relationship between MM-PHQ-9 change and self-rated CGI-Improvement at follow-up (*r*(45) = −0.64, *P <* 0.001). The correlation was negative because a lower CGI-Improvement score indicates larger improvement; however, it shows that more improvement on the MM-PHQ-9 was associated with more improvement on the CGI-Improvement. For reference, the correlation between the QIDS-SR-16 follow-up score and CGI-Improvement score was also moderate (*r*(44) = −0.56, *P* < 0.001).

### Internal consistency

Internal consistency of the MM-PHQ-9 was assessed for the total sample, and participants with MDD and controls separately at baseline. Cronbach's alpha for the internal consistency of the MM-PHQ-9 for the total sample was excellent (*α*(101) = 0.93). Inter-item correlations were at a moderate level, which indicated that items were measuring the same construct but were not too high to be redundant on an individual item basis. Cronbach's alpha was lower for the MDD sample than for the total sample, but was still good (*r*(78) = 0.76). Inter-item correlations were lower than for the total sample and did not exceed 0.6. Some items appeared to have no relationship, such as item 1 (‘Little interest or pleasure in doing things’) and item 5 (‘Worrying that you have done something wrong’), which correlated at *r* = −0.01. Cronbach's alpha in the control sample was good (*α*(23) = 0.78), and similar to that of the MDD sample. There was a similar relationship between items 1 and 5 for controls and the MDD sample, in that the items were unrelated. There was no indication that deletion of any of items would substantially improve the Cronbach's alphas of the scales.

### Principal component analysis

Principal component analysis was conducted on the MM-PHQ-9 item scores provided by 73 participants from ADeSS studies 1 and 2. A Kaiser–Meyer–Olkin test revealed the data to be appropriate for factor analysis (0.91), and a Bartlett's test of sphericity showed adequate homogeneity of variance (*χ*^2^(36) = 690.60, *P* < 0.001). The component matrix is included in Table 4, and the pattern, structure and component correlation matrices are included in Supplementary Tables 1–3). The component matrix shows all items to load above 0.4 onto factor 1, which had an Eigenvalue of 5.81 and explained 64.6% of the total variance, indicating a common construct, whereas factor 2 added an additional 11.4% of explained variance. The pattern and structure matrices (Supplementary Tables 1 and 2) showed that factor 1 was most strongly associated with symptoms that are thought to be an expression of overgeneralised self-blame,^28^ such as hopelessness, worries about one's mistakes and low self-worth. In contrast, factor 2 was inversely related with symptoms and factor 1 (Supplementary Table 3). Interestingly, the strongest negative associations of factor 2 were found with lack of interest/pleasure, energy and concentration.

**Table 4:** Principal component matrix loadings of MM-PHQ-9 items

Component	1	2
Little interest or pleasure	0.794	−0.431
Feeling down or depressed	0.888	−0.091
Feeling hopeless	0.868	0.225
Feeling tired or having little energy	0.815	−0.443
Worrying that you have done something wrong	0.767	0.429
Feeling bad about yourself	0.837	0.287
Trouble concentrating on things	0.766	−0.426
Feeling nervous, anxious or on edge	0.823	0.157
Thoughts that you would be better off dead or hurting yourself	0.650	0.333

Oblimin rotation with Kaiser normalisation was applied. MM-PHQ-9, Maudsley-modified Patient Health Questionnaire-9.

## Discussion

Our results show that the MM-PHQ-9 exhibited good concurrent validity when compared with standard self-rated measures of depression. The MM-PHQ-9 is designed to be used repeatedly by individuals to indicate change in symptoms over time; hence, the change in MM-PHQ-9 from baseline to follow-up was compared with a standard measure of change, the CGI-Improvement, which demonstrated a moderate relationship. The measure demonstrated 100% specificity and positive predictive values, and almost 100% sensitivity and negative predictive values, when differentiating the MDD and control samples according to a MM-PHQ-9 cut-off score of 10. This indicates the measure's potential to differentiate those with significant symptoms of depression more likely to be associated with MDD from those with mild symptoms observed in the general population, but this result needs to be interpreted with caution as it could have been driven by sample selection biases, and needs to be investigated in a naturalistic sample to probe its generalisability to clinical settings.

Although the QIDS-SR-16 indicated a larger effect size for detecting change in symptoms, the MM-PHQ-9 was able to detect changes at *d* = 0.41, which is approaching a moderate effect size. Further validation studies are needed to determine whether sensitivity to change in response to antidepressant treatment is comparable to that of the QIDS-SR-16, which cannot be determined in our sample, which consisted of a mix of different types of treatment and naturalistic outcomes. It will be important to determine whether the MM-PHQ-9's greater reliance on symptom pervasiveness compared with the QIDS-SR-16's focus on severity may make the latter more sensitive to change.

The MM-PHQ-9 had good internal consistency for the MDD sample, indicating that the items can reliably assess depressive symptoms. The newly added items to assess hopelessness, self-blame and anxiety correlated with other items at a level comparable to the original PHQ-9 items, implying that the new items are relevant for depression severity. However, when the sample was split into MDD and healthy control participants, the newly added item 5 (‘Worrying that you have done something wrong’) correlated negatively with original items 1 (‘Little interest or pleasure in doing things’) and 4 (‘Feeling tired or having little energy’). In contrast, item 8 (‘Feeling nervous, anxious or on edge’) correlated positively with all original PHQ-9 items, which indicates that anxiety is indeed strongly associated with other core symptoms.

Interestingly, this partial dissociation of self-blame-related and anhedonia-related items is also reflected in the two factors derived from our principal component analysis, which revealed one main factor, interpreted as a common depression factor as it reflects variance common to all items but is most strongly associated with overgeneralised self-blame-related items, and a second factor that is inversely related and reflects interest, pleasure and motivation more specifically. A previous body of research has found evidence for a two-factor structure of PHQ-9 items, namely a somatic and a non-somatic factor.^13,29–31^ As we removed somatic items when creating the MM-PHQ-9 it was expected that no somatic factor would remain, which is in line with our findings.

We also demonstrated that the mobile app version of the MM-PHQ-9 had good concurrent validity with the paper/electronic survey version. Digital mental health is rapidly developing, providing opportunities for individuals to independently monitor their symptoms in a quick and easy manner. Hence, it is key for research to compare accuracy of results via smartphone and standard presentation before utilising technology for depression rating scales and for the MM-PHQ-9 to be validated as suitable for further e-health applications.

### Limitations and future research

Despite indications of good concurrent validity and reliability, there are several limitations that must be acknowledged. The research is limited by variable inclusion criteria across the different data-sets. ADeSS study 1 specified a diagnosis of MDD as its inclusion criteria, and the NeuroMooD study specified a diagnosis of recurrent MDD, both according to the DSM-5. However, ADeSS study 2 did not recruit a clinically diagnosed sample, instead requiring a PHQ-9 score of ≥15. Despite the lack of diagnostic inclusion criteria for the total sample limiting the clinical application of the MM-PHQ-9, the range of severity and chronicity present in this study can be beneficial for creating a measure that captures the full spectrum of depressive severity. Measures of depression often have too limited ranges to capture those with both non-severe and severe depression.^14^ Therefore, a universal measure such as the MM-PHQ-9, which has been tested on individuals with and without a current diagnosis or not fully remitted, could improve the accuracy of self-reported symptoms without the need for separate assessments for different severities of depression.

Similarly, the ranging study designs meant that the studies were not homogenous in treatment types and durations. Participants from ADeSS studies 1 and 2 were taking antidepressants at baseline (or had in past 2 months), whereas NeuroMooD study participants were mixed in antidepressant use. Participants from ADeSS studies 1 and 2 were permitted to undergo psychotherapy during study participation, but NeuroMooD study participants were not. ADeSS study 1 participants had their antidepressant treatment reviewed within 2 weeks of baseline and potentially changed to another antidepressant or higher dose, whereas study 2 participants were not reviewed, and NeuroMooD study participants were required to remain on the same antidepressant and dose and received neurofeedback. Because of the potentially mixed impact on response of the different treatments, the sensitivity to change findings of *d* = 0.41 for the MM-PHQ-9 should be viewed tentatively. However, the comparative QIDS-SR-16 sensitivity to change of *d* = 0.61 provides some reassurance that the MM-PHQ-9 captured real change in symptoms over time.

Another limitation arising from the various data sources was a small sample size resulting from not all studies administering all measures of interest. This limits the confidence that can be had in some of the findings, particularly for the small sample on whom mobile app delivery of the MM-PHQ-9 was tested. Although there are twice as many women than men with MDD, both our clinical and control samples comprised over 80% women. Additionally, the result of our factor analysis may have been biased by specific characteristics of our sample, and will require replication in a sample that also includes a broader range of patients with MDD at different stages of recovery. Conversely, many other participant characteristics were widely heterogenous, such as age and depression subtype. Although such heterogeneity is beneficial for capturing a pragmatic sample to whom findings can be generalised, this approach lacks the specificity required to understand differences in response to a new measure by different subgroups. Future research should also probe the influence of item wording, which has been shown to be suboptimal for the standard PHQ-9 from the patient perspective.^32^

The mobile app used for data collection was developed as a pilot study in the ADeSS. Because of app installation issues, initial mobile app scores could have been submitted up to approximately 2 weeks after baseline, which could have reduced the relationship between the scores at the two time points. This is particularly important for ADeSS study 1 data, in which participants may have changed antidepressant within the 2-week period, although the effects of this would likely have a delayed effect. Future research should aim to compare app and paper MM-PHQ-9 scores for the same time point, to provide stronger alternate-form validity. In summary, our data provides preliminary evidence that the MM-PHQ-9 is a valid and reliable outcome measure of core depressive symptoms. The data is freely available for use and further validation in larger samples.

## Data Availability

The anonymised data that support the findings of this study are available from the corresponding author, R.Z., upon reasonable request.

## Supplementary material

For supplementary material accompanying this paper visit http://dx.doi.org/10.1192/bjo.2021.953. click here to view supplementary material
