# Supplementary material for: Development and validation of the Maudsley Modified Patient Health Questionnaire (MM-PHQ-9)
Source: BJPsych Open. 2021 Jul 2;7(4):e123. doi: 10.1192/bjo.2021.953 (PMC8281039; doi:10.1192/bjo.2021.953)
Supplement: Supplementary file 1 [file bjosup.zip › S2056472421009534sup001.docx]

**SUPPLEMENTARY MATERIALS**

**Development and Validation of the**

**Maudsley Modified Patient Health Questionnaire (MM-PHQ-9)**

Phillippa Harrison^1^, Syndi Walton^1^, Diede Fennema^1^, Suqian Duan^1^, Tanja Jaeckle^1^, Kimberley Goldsmith^2^, Ewan Carr^2^, Mark Ashworth^3^, Allan. H. Young^1,4^, & Roland Zahn^1,4^

*^1^* *Department of Psychological Medicine, Centre for Affective Disorders, Institute of Psychiatry, Psychology & Neuroscience, King’s College London, London, SE5 8AZ, UK*

^2^ Department of Biostatistics and Health Informatics, Institute of Psychiatry, Psychology & Neuroscience, King’s College London, London, UK

^3^ School of Population Health and Environmental Sciences, King’s College London, London, UK

4 South London and Maudsley NHS Foundation Trust, London, UK

Corresponding author:

Dr Roland Zahn (see address above)

E-mail: roland.zahn@kcl.ac.uk

Phone: 0044-(0)20 7848 0348

Fax: 0044-(0)20 7848 0298

**Supplementary Methods**

***Diagnostic procedures***

A diagnosis of MDD was made using DSM criteria in studies: We used the Structured Clinical Interview for DSM5(1) in NeuroMooD and ADeSS studies 1 and 3, whereas ADeSS study 2 used a detailed online self-report-based assessment using a standard PHQ-9 score of at least 15, shown to be highly specific for a current MDE(2), in addition to a history of treated depression in primary care and exclusion of a bipolar disorder using a validated self-report instrument (score≥9, PPV=84% in primary care (3)) and alcohol abuse (4) using validated self-report questionnaires. We excluded drug abuse by modifying the patient health questionnaire alcohol questions(4) for drugs and used three previously validated questions for excluding schizophreniform disorders(5). Patients were also asked to self-report medical or neurological conditions as potential exclusions.

**Supplementary Tables**

**Table A| Pattern Matrix for Principal Component Analysis of MM-PHQ-9 Items**

| Component | 1 | 2 |
| --- | --- | --- |
| Little interest or pleasure | .00 | -.90 |
| Feeling down, or depressed | .44 | -.55 |
| Feeling hopeless | .78 | -.17 |
| Feeling tired or having little energy | .00 | -.93 |
| Worrying that you have done something wrong | .95 | .12 |
| Feeling bad about yourself | .83 | -.08 |
| Trouble concentrating on things | -.01 | -.88 |
| Feeling nervous, anxious or on edge | .68 | -.23 |
| Thoughts that you would be better off dead or of hurting yourself | .77 | .07 |

Loadings of Maudsley-modified PHQ-9 items onto each factor are reported.

**Table B| Structure Matrix for Principal Component Analysis of MM-PHQ-9 Items**

| Component | 1 | 2 |
| --- | --- | --- |
| Little interest or pleasure | .56 | -.90 |
| Feeling down, or depressed | .78 | -.82 |
| Feeling hopeless | .89 | -.65 |
| Feeling tired or having little energy | .58 | -.93 |
| Worrying that you have done something wrong | .87 | -.47 |
| Feeling bad about yourself | .88 | -.60 |
| Trouble concentrating on things | .54 | -.88 |
| Feeling nervous, anxious or on edge | .82 | -.65 |
| Thoughts that you would be better off dead or of hurting yourself | .73 | -.41 |

This shows the correlations between variables and factors.

**Table C| Component Correlation Matrix for Principal Component Analysis of MM-PHQ-9**

| Component | 1 | 2 |
| --- | --- | --- |
| Factor 1 | 1.00 | -.62 |
| Factor 2 | -.62 | 1.00 |

This shows the moderately negative correlations between the two factors.

**Table D| Coordinates of the Receiver Operating Curve**

| **Positive if ≥^a^** | **Sensitivity**  **(True Positive Rate)** | **1 – Specificity**  **(False Positive Rate)** |
| --- | --- | --- |
| -1.00 | 1.000 | 1.000 |
| 0.50 | 1.000 | 0.698 |
| 1.50 | 1.000 | 0.628 |
| 2.50 | 1.000 | 0.558 |
| 3.50 | 1.000 | 0.465 |
| 4.50 | 1.000 | 0.372 |
| 5.50 | 1.000 | 0.302 |
| 6.50 | 1.000 | 0.233 |
| 7.50 | 0.987 | 0.233 |
| 8.50 | 0.987 | 0.186 |
| 9.50 | 0.987 | 0.000 |
| 10.50 | 0.949 | 0.000 |
| 11.50 | 0.923 | 0.000 |
| 12.50 | 0.782 | 0.000 |
| 13.50 | 0.744 | 0.000 |
| 14.50 | 0.692 | 0.000 |
| 15.50 | 0.628 | 0.000 |
| 16.50 | 0.564 | 0.000 |
| 17.50 | 0.500 | 0.000 |
| 18.50 | 0.397 | 0.000 |
| 19.50 | 0.321 | 0.000 |
| 20.50 | 0.244 | 0.000 |
| 21.50 | 0.205 | 0.000 |
| 22.50 | 0.154 | 0.000 |
| 23.50 | 0.090 | 0.000 |
| 24.50 | 0.051 | 0.000 |
| 25.50 | 0.026 | 0.000 |
| 27.00 | 0.000 | 0.000 |
| Displays the coordinates of the Receiver Operating Characteristic (ROC) curve for Maudsley-modified PHQ-9 scores. *The smallest cut-off value is the minimum observed test value-1, and the largest cut-off value is the maximum observed test value + 1. All the other cutoff values are the averages of two consecutive ordered observed test values. The table shows that the optimal cut-off point for MDD vs. control participants was ≥ 9.5 because this captured the highest true positive rate combined with the lowest false positive rate. As half points are not allowed on the questionnaire, this means that anyone with a score of 10 or higher would have been classified as MDD with a 0% false positive rate and 98.7% true positive rate in our sample. | | |

**Supplementary Figures**

**

**

**Supplementary Figure 1**| Results of the Receiver Operating Characteristic Curve (ROC) analysis including all 78 MDD and 43 Control participants. The black line represents a chance relationship between false positive (1-specificity) and true positive (sensitivity) rates. The blue line represents classification results using different values of the Maudsley-Modified PHQ-9 in our sample. The area under the ROC curve was 0.997 (standard error=.003, asymptotic p-value <.0001, 95% Confidence Interval: .991 to 1.000). For details of each potential cut-off score, please see Table D.

**Supplementary References**

1. First M, Williams J, Karg R, Spitzer R. User’s Guide for the Structured Clinical Interview for DSM-5 Disorders, Research Version (SCID-5-RV). Arlington, VA: American Psychiatric Association; 2015.

2. Manea L, Gilbody S, McMillan D. Optimal cut-off score for diagnosing depression with the Patient Health Questionnaire (PHQ-9): a meta-analysis. CMAJ. 2012;184(3):E191-6.

3. Kessler RC, Akiskal HS, Angst J, Guyer M, Hirschfeld RMA, Merikangas KR, et al. Validity of the Assessment of Bipolar Spectrum Disorders in the WHO CIDI 3.0. J Affect Disord. 2006;96(3):259-69.

4. Spitzer RL, Kroenke K, Williams JW, and the Patient Health Questionnaire Primary Care Study G. Validation and utility of a self-report version of prime-md: The phq primary care study. JAMA. 1999;282(18):1737-44.

5. Lythe KE, Moll J, Gethin JA, Workman C, Green S, Lambon Ralph MA, et al. Self-Blame-Selective Hyperconnectivity Between Anterior Temporal and Subgenual Cortices and Prediction of Recurrent Depressive Episodes. JAMA Psychiatry. 2015;72(11):1-8.
