# Supplementary material for: Development and validation of the Maudsley Modified Patient Health Questionnaire (MM-PHQ-9)
Source: BJPsych Open. 2021 Jul 2;7(4):e123. doi: 10.1192/bjo.2021.953 (PMC8281039; doi:10.1192/bjo.2021.953)
Supplement: Supplementary file 1 [file bjosup.zip › S2056472421009534sup002.docx]

**Maudsley-modified PHQ-9**

| **Over the last week, how often have you been bothered by any of**  **the following problems?** | | **Not at all** | **Some days** | **More than half the days** | **Every  day** |
| --- | --- | --- | --- | --- | --- |
| 1 | Little interest or pleasure in doing things | 0 | 1 | 2 | 3 |
| 2 | Feeling down, or depressed | 0 | 1 | 2 | 3 |
| 3 | Feeling hopeless | 0 | 1 | 2 | 3 |
| 4 | Feeling tired or having little energy | 0 | 1 | 2 | 3 |
| 5 | Worrying that you have done something wrong | 0 | 1 | 2 | 3 |
| 6 | Feeling bad about yourself — or that you are a failure | 0 | 1 | 2 | 3 |
| 7 | Trouble concentrating on things, such as reading the newspaper or watching television | 0 | 1 | 2 | 3 |
| 8 | Feeling nervous, anxious or on edge | 0 | 1 | 2 | 3 |
| 9 | Thoughts that you would be better off dead or of hurting yourself  in some way | 0 | 1 | 2 | 3 |
|  |  | MM-PHQ-9 total sum score | | |  |

**Notes on changes from original Patient Health Questionnaire (PHQ-9)**

- Questions 2 and 3 were separated.
- Somatic symptoms (sleep, appetite) were omitted.
- Psychomotor activity was omitted.
- Question 5 was added to ask for self-blaming emotions and was previously validated (1) to detect self-blaming emotions in 60% of patients with MDD. This dissociates from low self-worth and therefore question 6 was simplified.
- Intervals were changed from biweekly to weekly in keeping with other depression scales used for detecting response (QIDS-SR-16(2)) and to improve memory for the period in question as well as improving sensitivity to change. This necessitated changing the wording of the scale anchors slightly to “Some days” instead of “Several days” and “Every day” instead of “Nearly every day”.

**Scoring and purpose**

This scale was developed for the Antidepressant Advisor study (ADeSS) (3) and is integrated into the MooDoC mobile app (<https://play.google.com/store/apps/details?id=com.allocmodulo&hl=en_IN>). It was designed for tracking symptoms rather than for diagnostic purposes. Although, the scoring range is the same as the original PHQ-9 scale (0-27), the scores cannot be directly compared. In our initial study, our control group without major depressive disorder did not score above 9, but this cannot be used for diagnostic purposes, only as an indication of the potential clinical relevance of scores above 9, subject to further validation.

**Acknowledgements**

We are grateful for the original PHQ-9 screener(4, 5), which was developed by Drs. Robert L. Spitzer, Janet B.W. Williams, Kurt Kroenke and colleagues, with an educational grant from Pfizer Inc.

Validation of the MM-PHQ-9 represents independent research partly funded by the National Institute for Health Research (NIHR) research for patient benefit scheme (grant reference: PB-PG-0416-20039) and by the National Institute for Health Research (NIHR) Biomedical Research Centre at South London and Maudsley NHS Foundation Trust and King’s College London. The views expressed are those of the authors and not necessarily those of the NHS, the NIHR or the Department of Health and Social Care.

**References**

1. Zahn R, Lythe KE, Gethin JA, Green S, Deakin JF, Young AH, et al. The role of self-blame and worthlessness in the psychopathology of major depressive disorder. J Affect Disord. 2015; 186: 337-41.

2. Rush AJ, Trivedi MH, Ibrahim HM, Carmody TJ, Arnow B, Klein DN, et al. The 16-Item Quick Inventory of Depressive Symptomatology (QIDS), clinician rating (QIDS-C), and self-report (QIDS-SR): a psychometric evaluation in patients with chronic major depression. Biological psychiatry. 2003; 54(5): 573-83.

3. Harrison P, Carr E, Goldsmith K, Young AH, Ashworth M, Fennema D, et al. Study protocol for the antidepressant advisor (ADeSS): a decision support system for antidepressant treatment for depression in UK primary care: a feasibility study. BMJ Open. 2020; 10(5): e035905.

4. Spitzer RL, Kroenke K, Williams JW, and the Patient Health Questionnaire Primary Care Study G. Validation and utility of a self-report version of prime-md: The phq primary care study. JAMA. 1999; 282(18): 1737-44.

5. Spitzer RL, Williams JW, Kroenke K, et al. Utility of a new procedure for diagnosing mental disorders in primary care: The prime-md 1000 study. JAMA. 1994; 272(22): 1749-56.
